# The Influence of Spirituality in the Care of Patients with Advanced Chronic Illnesses and at the End of Life: An Integrative Review

**DOI:** 10.1007/s10943-025-02543-9

**Published:** 2026-01-12

**Authors:** José M. Pérez-Jiménez, Patricia Bonilla Sierra, Rocío de-Diego-Cordero

**Affiliations:** 1 Department of Nursing. Schools of Nursing, Physiotherapy, and Podiatry, C/Avenzoar 9, 41006 Seville, Spain; 2 Department of Anesthesiology and Resuscitation, University Hospital Virgen Macarena, 41009 Seville, Spain; 3 Department of Health Sciences. Private, Technical University of Loja, París S/N y Praga, 110104 Loja, Ecuador; 4 Inter-university Doctoral Program in Health Sciences, University of Seville, C/Avenzoar 9, 41006 Seville, Spain

**Keywords:** Spirituality, Religion, Spiritual well-being, Palliative care, Chronic illness

## Abstract

Spirituality is a central yet often overlooked component of care, particularly for people facing advanced chronic illness or approaching the end of life. This integrative review examined evidence on how spiritual care influences emotional, existential, and quality of life outcomes, and identified factors that facilitate or hinder its integration into clinical practice. We searched EMBASE, PubMed, Scopus, Web of Science, BvS, Cochrane, and gray literature for peer-reviewed studies published between 2015 and 2025. Inclusion criteria included adults (≥ 18 years) with advanced or terminal illness, as well as quantitative, qualitative, mixed-methods, and review designs, and publications in English, Spanish, or Portuguese. Twenty-four studies met eligibility criteria. Across all settings and diagnoses, higher spiritual well-being was consistently linked to lower anxiety and depression, greater hope and resilience, better health-related quality of life, and greater acceptance of death. Addressing spiritual needs, particularly meaning, belonging, reconciliation, and death preparation, reduced distress and enhanced dignity when integrated into Advance Care Planning. However, heterogeneity in spirituality definitions, limited professional training, and weak institutional support hindered consistent implementation. These findings underscore spirituality as a determinant of health that should be systematically assessed, taught, and incorporated into the care of patients with serious illness. A brief spiritual assessment, structured meaning-centered conversations with family inclusion, and Advance Care Planning that reflects patients’ beliefs and values are recommended.

## Introduction

Spirituality is increasingly recognized as a core dimension of person-centered healthcare, especially in the context of serious illness and end-of-life care, where it supports meaning-making, coping, and dignity (Puchalski et al., 2014, 2018). Beyond symptom control, well-being at this stage requires care aligned with patients’ values, beliefs, and goals, delivered within ethical and clinical standards (Carr & Luth, 2019; Inbadas, 2017; López-Tarrida et al., 2021). Advance care planning (ACP) further operationalizes these commitments by honoring preferences during incapacity and reducing uncertainty for patients and families (Rietjens et al., 2017; Sudore et al., 2017). Conceptually, spirituality reflects a dynamic human search for connection, purpose, and transcendence expressed through beliefs and practices that have measurable effects on physical, psychological, and social health (Puchalski et al., 2014; Sena et al., 2021).

Empirically, spirituality and religiosity relate to better psychological adjustment, reduced anxiety and hopelessness, and resilience in chronic and terminal illness (Avcı & Çavuşoğlu, 2024; Best et al., 2023; Lifshitz et al., 2019; McClain et al., 2003; Reissmann et al., 2021). However, patients continue to report unmet spiritual needs alongside loneliness and distress, underscoring implementation gaps (Kurtgöz & Genç, 2024). Moreover, much of the literature privileges individualistic framings and underexamines cultural-historical contexts and system-level delivery strategies; controlled evaluations of spiritual care interventions also remain limited (Avcı & Çavuşoğlu, 2024; Badanta et al., 2022; Best et al., 2023; Glyn-Blanco et al., 2023; Inbadas, 2016). Integrating spirituality with ACP may help align decisions with what matters most, while fostering compassionate clinician-patient relationships at the end of life (Grabenweger et al., 2024; Kang et al., 2024; Stelcer et al., 2023).

Spirituality plays a fundamental role in coping, finding meaning, and preserving dignity in advanced illness and at the end of life; however, its application in palliative care remains limited. Confusion, often in conceptual definitions, and limited professional training hinder its routine incorporation into care. Therefore, this integrative review aims to answer the following research question: How does spirituality, in relation to attitudes toward death, well-being, quality of life, and religiosity, influence the care and well-being of adults with advanced chronic illnesses or nearing the end of life, within the context of palliative care?

## Methods

### Study Design

An integrative literature review (Toronto, 2020; Whittemore & Knafl, 2005) was conducted, encompassing qualitative, quantitative, and mixed-methods studies, as well as non-randomized clinical trials, to deepen the understanding of how spirituality influences the care of patients with advanced chronic illnesses and at the end of life. To conduct the study, the authors followed five essential stages of an integrative review: (a) problem identification, (b) literature search, (c) data evaluation, (d) data analysis, and (e) presentation of results (Souza et al., 2010).

### Methods and Search Strategy

To ensure methodological rigor, this review was conducted in accordance with PRISMA guidelines (Tricco et al., 2018). A specialized librarian from the University of Seville collaborated in designing the search criteria, which were informed by expert consultation and previous studies on spirituality and religiosity (*R*/*S*) in patients with chronic illnesses and within palliative care contexts.

**Fig. 1:**
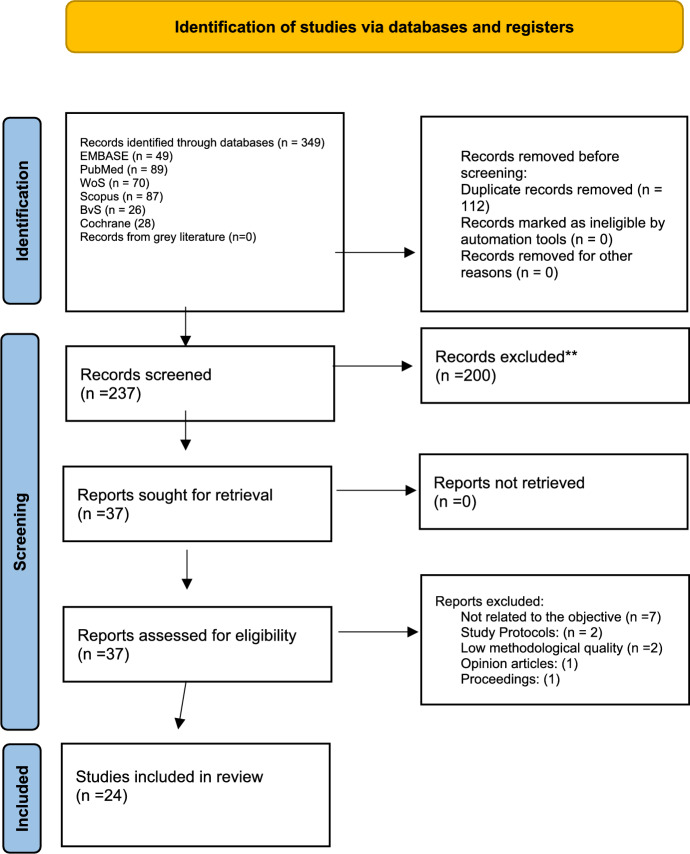
Flowchart for Paper Identification, Screening, and Inclusion

A comprehensive literature search was conducted across the electronic databases PubMed, Scopus, EMBASE, Web of Science, the Virtual Health Library (VHL), and the Cochrane Library, complemented by a review of gray literature via OpenGrey. All references were managed using Zotero 7 software. The search spanned 2015–2025; we chose 5 years to limit our findings to the most up-to-date peer-reviewed scientific literature, which may reflect the growing volume of work in the field of spirituality research. To identify relevant literature for this review, a comprehensive literature search was conducted in October 2024, using all fields in six databases. These databases were chosen because they offer several key methodological advantages, including higher quality, reliability, and replicability. To keep the integrative review up to date, a second search was conducted in March 2025. The strategy was designed to address the research question, focusing on the influence of spirituality on well-being in advanced illness and end-of-life care. Several synonyms for each keyword group were identified using MeSH terms across the six databases, complemented by additional related terms obtained through web searches and previously published literature, resulting in a final list of 14 search terms. The search strategy, which incorporated MeSH terms such as “attitude to death,” “well-being,” “quality of life,” “spirituality,” “religion,” “palliative care,” and “chronic diseases,” was verified following established guidelines for electronic search methods (McGowan et al., 2016) to ensure completeness. Boolean operators were applied to refine combinations and optimize retrieval. In addition, preliminary searches were conducted in each database to confirm the relevance and sensitivity of the selected terms, acknowledging that each digital platform operates with distinct search engine requirements. The selection process followed PRISMA 2020 guidelines (Page et al., 2021), ensuring methodological transparency and reproducibility. After removing duplicates, studies meeting the predefined inclusion criteria were retained, as illustrated in the PRISMA flow diagram (Fig. 1).

A total of 349 records were initially identified through database searches and gray literature sources. After removing 112 duplicates, 237 titles and abstracts were screened. Of these, 37 full-text articles were assessed for eligibility, and 11 were excluded because they did not meet the inclusion criteria, primarily due to not explicitly examining spirituality or spiritual care in relation to well-being, quality of life, or end-of-life outcomes. An additional two studies were excluded due to methodological limitations. Finally, 24 studies met all inclusion criteria and were incorporated into the final synthesis of this integrative review.

### Inclusion and Exclusion Criteria

The PRISMA selection process was used to screen publications and determine their eligibility. All studies were evaluated according to predefined inclusion and exclusion criteria, guided by the research question and the purpose of this integrative review. Studies were included if they examined the influence of spirituality or spiritual care on the well-being of adults (≥ 18 years) with advanced chronic illness or at the end of life; were peer-reviewed publications presenting original data from primary or secondary research; employed quantitative, qualitative, or mixed-methods designs; were published between 2015 and 2025; and were available in English, Spanish, or Portuguese. Studies in which spirituality was not addressed conceptually or clinically were excluded; for example, when it appeared only as a demographic variable or when interventions lacked an explicit spiritual component. Studies focused exclusively on evaluating specific tools for measuring spirituality or religiosity were also excluded, as were studies focusing on pediatric populations, non-peer-reviewed materials (such as opinion pieces, protocols, or conference abstracts), manuscripts with insufficient methodological detail or unvalidated instruments, and publications for which the full text was unavailable. Gray literature was considered only when it had institutional support and met rigorous methodological standards (Paez, 2017). This process ensured the relevance, coherence, and methodological robustness of the evidence synthesized.

### Data Extraction

Before beginning data extraction, a trained librarian and a professor with expertise in health research reviewed the search terms, databases, and eligibility strategy to ensure consistency and comprehensiveness. In the first phase, duplicates were removed; in the second, titles and abstracts were reviewed; and in the third, the full texts of potentially eligible studies were reviewed to confirm they met the inclusion criteria.

One researcher independently conducted the data extraction process, while a second reviewer verified the accuracy and completeness of the information. The following data were extracted from each study: authors, year of publication, country, sample design and characteristics, indicators of spirituality or faith, health and well-being outcomes, theoretical or conceptual frameworks, and main findings (Table 1). Data were recorded on a standardized form refined during the calibration phase to ensure inter-rater reliability (Toronto, 2020).

**Table 1:** Characteristics and main findings of included studies

References, country	Study objective	Study design and sample characteristics	Data collection and instruments	Main findings
Testoni et al. (2018) Italy	To examine the role of meaning in life and representations of death in psychological distress, anxiety, and depression among cancer patients	Cross-sectional study in two phases. 219 healthy participants and 30 cancer patients completed questionnaires on meaning, death perception, anxiety, depression, and distress	Personal Meaning Profile, Testoni Death Representation Scale, Hospital Anxiety and Depression Scale, Distress Thermometer	Greater meaning in life was associated with lower distress, anxiety, and depression. Viewing death as a transition rather than annihilation reduced psychological suffering. Faith in God strengthened life meaning, while absence of religious beliefs was linked to higher anxiety. The study supports incorporating meaning-centered strategies in palliative care
Moestrup and Hvidt (2016) Denmark	To analyze how Danish hospice patients reflect on spirituality and religion, and how this reflection influences their experience and well-being at the end of life	Qualitative study. 17 patients and 9 family	Semi-structured interview guide with open-ended questions, field notes, systematic documentation of observations, direct quotations, and categorization using the ‘Knowing, Doing, and Being’ model	Despite living in a secular society, patients expressed a need for transcendence and meaning-making. Their spirituality, uncertain and unstructured, was manifested through knowledge, action, and belonging. While some found comfort in faith, others experienced religious struggle. Spiritual care in hospices was key to coping with the end of life, even in secular contexts
Mesquita et al. (2017) Brazil	To synthesize existing evidence on the spiritual needs of cancer patients in palliative care	Integrative literature review. 6 primary studies evaluating a total of 1,469 patients		Eight key spiritual needs were identified: finding meaning in life, understanding the illness, connecting with others, accessing spiritual practices, maintaining holistic well-being, discussing death, making the most of remaining time, and preserving autonomy
Masterson et al. (2018) USA	To examine the prevalence and common themes of unfinished business and its association with psychological distress in patients with advanced cancer	Randomized controlled trial. 223 patients with advanced cancer	Unfinished Business Questionnaire, Hopelessness Assessment in Illness, McGill Quality of Life Questionnaire, Hospital Anxiety and Depression Scale, Schedule of Attitudes Toward Hastened Death, Life Attitude Profile-Revised, Functional Assessment of Chronic Illness Therapy–Spiritual Well-Being Scale (FACIT-Sp)	72% of advanced cancer patients reported unfinished business, and 45% experienced significant distress, linked to higher anxiety, hopelessness, and lower sense of meaning. Existential disconnection and emotional distress increased desire for hastened death. Unfinished business was related to unmet goals and unresolved conflicts
Krikorian et al. (2020) Colombia	To explore the conceptions of a good death from the patients’ perspective	Systematic literature review. 29 publications		Spirituality and religiosity supported end-of-life patients by reducing anxiety, providing emotional comfort, and fostering acceptance of death. In various cultures, such as Mexican–American, these beliefs were key to experiencing a good death, promoting peace and meaning in the final moments
Guerrero-Torrelles et al. (2017) Spain	To identify and describe interventions that promote meaning in life in patients with advanced illnesses by evaluating the context, mechanisms, and outcomes of such interventions	Systematic literature review. 12 publications		Spirituality in the care of chronically ill patients had a positive impact on their well-being, promoting purpose, quality of life, and optimism, while reducing distress, anxiety, and hopelessness. Meaning-centered interventions highlighted the importance of the patient-therapist relationship in exploring sources of meaning and strengthening emotional connection
Delgado-Guay et al. (2016) USA	To evaluate the frequency, intensity, and correlations of spiritual pain in patients with advanced cancer attending a palliative care clinic	Cross-sectional observational study. 292 patients with advanced cancer	Edmonton Symptom Assessment Scale, modified to include a measure of spiritual pain	Spiritual care at the end of life improved patient satisfaction, quality of life, and facilitated treatment decision-making. It also promoted personal growth and more effective communication with clinicians, contributing to a more holistic and compassionate care
Byrne and Morgan (2020) New Zealand	To assess the prevalence of religiosity, death anxiety, and hope among community hospice patients in their final six months of life, and to explore whether religiosity acts as a protective factor against death anxiety	Cross-sectional study. 22 patients	Religious Commitment Inventory, Death and Dying Distress Scale, Hope Herth Index	Spirituality influenced end-of-life care primarily through connection with loved ones and the recollection of meaningful moments. Although organized religion was not a primary source of hope in this secular context, intrinsic religiosity was associated with greater well-being
Broadhurst and Harrington (2016) Australia	To investigate the meaning of hope for palliative care patients and examine the themes that foster hope in this population	Systematic review. 15 articles	Review of qualitative and mixed-method studies; specific tools not detailed	Spirituality and faith in God were key sources of hope in palliative care, facilitating adaptation to illness and acceptance of death. Hope evolved from expectations of cure to peace and legacy, reinforced by connection with others and pain control. These findings highlight the importance of spiritual care in improving quality of life and reducing existential suffering
Wisesrith et al. (2021) Thailand	To investigate the spiritual needs of Thai patients with terminal cancer in order to improve comprehensive care that allows them to experience greater well-being in the final stage of life	Cross-sectional study. 322 participants	Spiritual Needs Scale	The most important spiritual needs included preparation for death, meaning in life, and the opportunity to achieve meaningful goals. Patients with strong spiritual grounding and family support showed higher levels of well-being. Appropriate spiritual care contributed to illness acceptance, reduced suffering, and improved quality of life in the final stage
Meier et al. (2016) USA	To review the literature to identify unmet needs and encourage dialogue about death	Systematic literature review. 36 studies		Spirituality was essential to a "good death" for 65% of patients, and it was associated with emotional well-being, acceptance of death, and reduced suffering. Integrating spiritual support into palliative care improved quality of life and facilitated a dignified death
Kukla et al. (2022) Germany	To analyze how individuals over the age of 80 or with terminal illnesses cope with the end of life, and to examine the impact of this experience on their overall well-being. Additionally, the study aims to identify personal strategies and support needs that contribute to appropriate and compassionate care during the final stage of life	Qualitative study. 20 participants	Semi-structured interview guide, audio recordings, field notes	Spirituality helped older adults and patients with terminal illnesses cope with the end of life by providing relief, a sense of security, and purpose. Faith and personal reflection reduced fear of death; however, access to spiritual support remains limited, underscoring the need to integrate it into palliative care
Kruizinga et al. (2019) Netherlands	To analyze the effect of a structured interview focused on life events and goals on quality of life and spiritual well-being in patients with advanced cancer	Randomized controlled trial. A total of 153 patients with incurable cancer were included, with 77 in the intervention group and 76 in the control group	European Organization for Research and Treatment of Cancer Quality of Life Questionnaire—Core Module. Palliative care (EORTC QLQ-C15-PAL), Functional Assessment of Chronic Illness Therapy–Spiritual Well-Being Scale 12 (FACIT-Sp12), Satisfaction with Life Scale, Hospital Anxiety and Depression Scale, Custom Evaluation Form (Patient Satisfaction)	Spirituality influenced the quality of life of patients with advanced cancer. Although the intervention did not show significant improvements, the experience of meaning and peace was associated with greater well-being and satisfaction, highlighting its importance in palliative care
Kruizinga et al. (2017) Netherlands	To explore the relationship between spiritual well-being, images of God, and attitudes toward death in patients with cancer receiving palliative care	Cross-sectional study. 52 patients with advanced-stage cancer	European Organization for Research and Treatment of Cancer—Spiritual Well-Being 32 (EORTC QLQ-SWB32)	The image of an Unknowable God (inaccessible or impossible to comprehend) was negatively associated with spiritual well-being in palliative care patients, while Personal and Non-Personal images of God showed no significant impact. Moreover, spiritual well-being extended beyond traditional religious beliefs, highlighting the need for a more inclusive approach in palliative care to enhance connection, peace, and emotional adjustment at the end of life
Bernard et al. (2017) Switzerland	To analyze the relationship between spirituality, meaning in life, desire for hastened death, and psychological distress in palliative care patients, and their impact on quality of life at the end of life	206 patients in palliative care	Schedule for Meaning in Life Evaluation, FACIT-Sp, Idler Index of Religiosity, Hospital Anxiety and Depression Scale, Schedule of Attitudes toward Hastened Death, Single-Item Quality of Life Scale	Spiritual well-being and meaning in life protected against psychological distress and reduced the desire for hastened death in palliative care patients. Quality of life was more strongly influenced by spirituality than by formal religiosity, with depression being the main factor negatively affecting it
Grossman et al. (2018) Australia, Canada, and the United States	To evaluate the effectiveness of psychological interventions for death anxiety in patients with advanced cancer	Systematic review of controlled clinical and observational studies		The study identified effective interventions for reducing death anxiety in patients with advanced cancer, particularly those focused on spirituality and meaning in life. Meaning-Centered Therapy and Short-Term Life Review were shown to enhance spiritual well-being, strengthen life meaning and faith, and reduce anxiety and the desire for hastened death
Klimasiński et al. (2022) Poland	To evaluate the level of spiritual distress and spiritual needs in patients with chronic illnesses in Poland, as well as their relationship with independent factors to improve spiritual care in clinical settings	Cross-sectional study. 204 patients with chronic illnesses	Ad hoc questionnaire developed by the researchers, based on validated scales such as, FACIT-Sp, and Spiritual Needs Questionnaire	More than half of the patients with chronic illnesses reported spiritual distress, which was associated with disease severity but not with religiosity. The need to find meaning and the practice of prayer were the most common spiritual demands. Faith as a coping resource was linked to greater spiritual needs, underscoring the importance of integrating spiritual support into clinical care to improve well-being and quality of life
Tirgari et al. (2022) Not specified	To evaluate spiritual well-being in patients with chronic illnesses	Systematic review and meta-analysis. Twenty-four studies were selected, including a total of 3,289 patients with chronic illnesses		Spiritual well-being in patients with chronic illnesses was moderate and associated with better quality of life, greater resilience, and lower emotional distress. Spirituality served as a protective factor, promoting a sense of meaning in life and reducing stress
Cilona et al. (2023) Italy	To review the influence of spirituality and religiosity in patients with heart failure, evaluating their impact on quality of life, depression, and cardiovascular outcomes	Systematic review of observational studies and clinical interventions. A total of 1,234 heart failure patients across seven studies (three observational and four interventional)		Spirituality in patients with heart failure was associated with better quality of life and lower levels of depression. Spiritual peace reduced mortality risk, and an intervention based on education and reflection improved overall well-being. While formal religiosity showed no significant impact, spiritual well-being did—highlighting the need to integrate spiritual support into clinical care
Wade et al. (2018) United States	To examine the relationship between religiosity, spirituality, and happiness in adults with neurological illnesses, determining their impact on psychological adjustment and quality of life	Cross-sectional study with multivariate analysis. 354 patients with neurological illnesses	Spiritual Well-Being Scale, Pemberton Happiness Index, Short Questionnaire to Assess Health-Enhancing Physical Activity, Wechsler Abbreviated Scale of Intelligence – 2nd Edition, Wechsler Test of Adult Reading, Depression Visual Analog Scale	Existential spirituality was associated with greater happiness in patients with neurological illnesses, whereas religiosity showed no significant impact on quality of life. Spirituality helped mitigate the effects of depression, highlighting its protective role in emotional well-being
Al-Ghabeesh et al. (2018) Jordan and the United Arab Emirates	To analyze the meaning of spirituality for patients with end-stage renal disease and its relationship with health outcomes and overall well-being	Systematic review. 33 studies		Spirituality in patients with end-stage renal disease improved quality of life, reduced depression, and facilitated treatment adaptation. It served as a coping mechanism, providing emotional strength, while formal religiosity showed no direct impact
Cluley et al. (2024) United Kingdom	To analyze how chronic illness, specifically end-stage kidney disease and hemodialysis treatment, affects religious practice, cultural identity, and the well-being of patients from ethnic minorities	Qualitative study with thematic analysis. 19 patients	Semi-structured interview guide, qualitative analysis software (NVivo/Atlas.ti), field notes	Chronic kidney disease and hemodialysis disrupted patients’ religious and cultural practices, affecting their identity and emotional well-being. Although faith served as a coping resource, the inability to fully practice it caused distress
García-Navarro et al. (2021) Spain	To identify the spiritual needs of patients at the end of life and explore how nursing professionals can provide effective support during this process	Qualitative study with a phenomenological approach. 7 patients and 10 healthcare professionals	Semi-structured interview guide designed and piloted prior to data collection, audio recordings, and field notes	Spirituality was key to the emotional well-being of patients at the end of life, providing meaning, hope, and serenity. However, healthcare professionals acknowledged a lack of training in this area
Rego et al. (2020) Portugal	To analyze the influence of spirituality on decision-making in palliative care patients and its relationship with emotional well-being and autonomy	Cross-sectional study. 95 outpatient oncology palliative care patients	Decisional Conflict Scale, FACIT-Sp, Sociodemographic Questionnaire, Semi-Structured Interview	Greater spiritual well-being in palliative care patients was associated with better quality of life, reduced uncertainty, and greater confidence in decision-making. Spirituality, linked to family and religion, reduced decisional conflict and improved satisfaction with care

The table describes the characteristics and findings of the included studies that examined the influence of spirituality on the care of patients with advanced chronic illness and at the end of life. This intergrative review did not involve an assessment of studies that utlized contaminated scales as this was beyond the aims of the research (refer Koenig & Carey, 2024)

FACIT-Sp, Functional Assessment of Chronic Illness Therapy–Spiritual Well-Being Scale; FACIT-Sp12, Functional Assessment of Chronic Illness Therapy–Spiritual Well-Being Scale 12; EORTC QLQ-SWB32, European Organization for Research and Treatment of Cancer—Spiritual Well-Being 32; EORTC QLQ-C15-PAL, European Organization for Research and Treatment of Cancer Quality of Life Questionnaire—Core Module. Palliative care

The review process followed international methodological guidelines: PRISMA 2020 for systematic reviews (Page et al., 2021), STROBE for observational studies (von Elm et al., 2014), CONSORT for clinical trials (Schulz et al., 2010), and SRQR for qualitative research (O’Brien et al., 2014), ensuring rigor, transparency, and reproducibility. Studies with a high risk of bias or low methodological quality were excluded from the final synthesis.

## Results

### Search Outcome

After full-text screening, the final sample comprised 24 studies (Table 1), four qualitative studies, two randomized controlled trials, eight systematic reviews, one integrative review, and nine observational studies (see Table 2).

**Table 2:** Study design of included studies

Study type	Number of studies
Qualitative	4
Randomized controlled trials	2
Systematic reviews	8
Integrative review	1
Observational	9
Total	24

### Study Characteristics

**Fig. 2:**
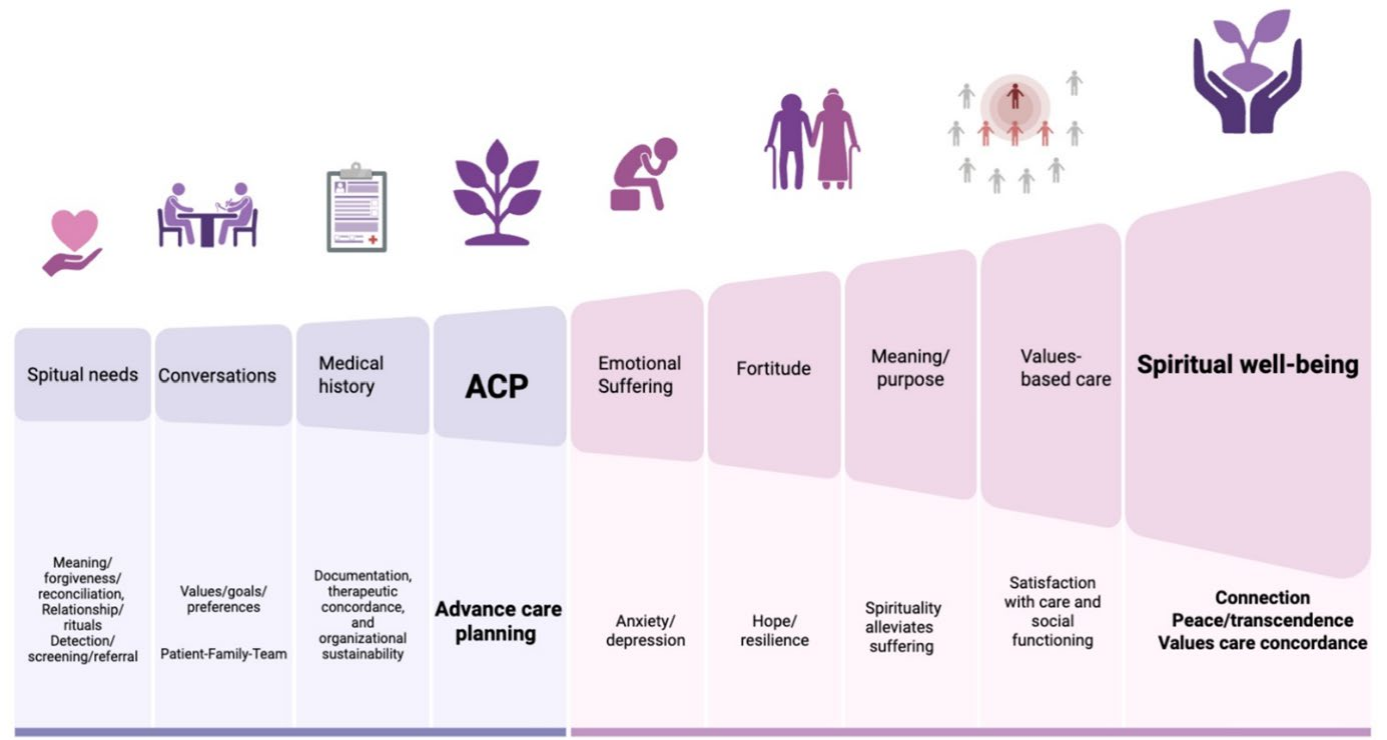
Model of Spiritual Well-Being Development in Advanced Illness

Most studies were published between 2017 and 2020 and conducted across multiple regions, with the USA being the most represented country. Sample sizes ranged from small qualitative designs (Guerrero-Torrelles et al., 2017) to large meta-analyses (Tirgari et al., 2022), reflecting methodological diversity. The findings are organized into five interconnected themes (emotional coping, spiritual well-being, quality of life, spiritual needs, and advance care planning), which together show how addressing spiritual dimensions not only alleviates suffering but also aligns care with what gives life meaning (see Fig. 2).

Model of Spiritual Well-Being Development in Advanced Illness: Spiritual well-being is strengthened through three operational and cyclical steps: (1) assessment of needs (meaning, belonging, reconciliation, and preparation for death); (2) structured and practical conversations focused on connection, transcendence, sense of purpose, and meaning/peace, with family inclusion; and (3) sustainable documentation within Advance Care Planning (ACP) to align care with the patient’s values and goals. This process reduces anxiety and depression, enhances hope and resilience, and improves quality of life, dignity at the end of life, and spiritual well-being, that is, the connection with oneself, with others, and with the transcendent.

#### Emotional Coping

Emotional coping in advanced illness is shown to be a profoundly human process, influenced by each person’s spiritual well-being and their capacity to find meaning and connection amidst their own experience of illness. Several studies have demonstrated that greater spiritual well-being was consistently correlated with lower anxiety and depression, better emotional balance, and greater peace in people facing cancer, heart failure, or life-limiting illnesses (Cilona et al., 2023; Kruizinga et al., 2019). Conversely, when spiritual needs, particularly for meaning, reconciliation, and preparation for death, were not met, distress intensified and coping diminished (Wisesrith et al., 2021). Hope and resilience were strengthened when clinical interactions addressed connection, transcendence, legacy, and purpose. Reviewing life, values, rituals, and family involvement reduced distress and increased hope (Wisesrith et al., 2021), enabling patients to cope with uncertainty with greater acceptance and psychological stability. Meaning-focused interventions further strengthened adaptation by reinforcing purpose and coherence, reducing the desire for hastened death, and promoting emotional balance (Bernard et al., 2017; Grossman et al., 2018).

#### Spiritual Well-Being

Spiritual well-being functioned as a central mechanism linking spirituality to clinical and psychosocial outcomes. The included studies demonstrated that the core dimensions of Meaning/Peace, Connection, and Transcendence protected emotional health, improved coping, and strengthened dignity at the end of life. Connection with oneself, others, nature, or the transcendent promoted the preservation of identity and relational security (Cilona et al., 2023; Moestrup & Hvidt, 2016), while disconnection increased suffering. Inner peace emerged as a therapeutic element strongly associated with life quality and satisfaction (Kruizinga et al., 2019), while perceiving the divine as distant or "unknowable" predicted lower spiritual well-being (Kruizinga et al., 2017). Interventions that promote meaning, reconciliation, and life fulfillment, such as guided conversations, reflective practices, and family involvement, reduced psychological distress and promoted dignity and acceptance (Klimasiński et al., 2022; Testoni et al., 2018; Wisesrith et al., 2021).

#### Health-Related Quality of Life

The influence of spirituality on HRQoL was evident in the physical, psychological, social, and spiritual domains. Several studies consistently demonstrated that greater spiritual well-being and coherence between care and patient values predicted better quality of life and reduced decision conflicts in oncology, heart failure, and palliative care settings (Cilona et al., 2023; Rego et al., 2020). Meaning and purpose served as pathways through which spirituality improved overall well-being and symptom experience, contributing to greater life satisfaction and autonomy in decision-making (Kruizinga et al., 2019; Testoni et al., 2018). Addressing spiritual distress improved communication, clarified preferences, and facilitated acceptance at the end of life (Delgado-Guay et al., 2016; García-Navarro et al., 2021). Likewise, honoring personal values and cultural or family priorities promoted dignity, improved psychosocial functioning, and strengthened relationships (Krikorian et al., 2020; Wisesrith et al., 2021).

#### Spiritual Needs

Spiritual needs represented the starting point of the spiritual well-being cycle, as their recognition guided significant clinical conversations and interventions. Studies revealed four consistent areas of need: meaning/purpose, reconciliation/forgiveness, belonging/connection, and meaningful practices or rituals. By exploring and addressing these needs, patients experienced less anxiety and hopelessness, better emotional adjustment, and greater acceptance of mortality (Masterson et al., 2018; Testoni et al., 2018). Meaning-focused interventions strengthened resilience and spiritual well-being (Grossman et al., 2018; Guerrero-Torrelles et al., 2017; Tirgari et al., 2022), while relational support, family involvement, and opportunities to maintain spiritual or cultural practices fostered comfort, continuity, and identity (Al-Ghabeesh et al., 2018; Cluley et al., 2024; Mesquita et al., 2017; Wade et al., 2018; Wisesrith et al., 2021). Failure to identify these needs was associated with greater distress, a desire to hasten death, and worse emotional outcomes (Bernard et al., 2017; Delgado-Guay et al., 2016). Therefore, systematic detection and timely referral within palliative care teams emerged as essential components of person-centered care (Rego et al., 2020).

#### Advance Care Planning (ACP)

ACP emerged as the clinical and organizational mechanism that allows for the transformation of patients’ values, beliefs, and priorities into concrete decisions throughout the care process. Unlike other dimensions of spirituality, which are oriented toward meaning, connection, or emotional coping, ACP primarily acts as an operational bridge between what matters to the person and the actions of the healthcare team. Studies have shown that when ACP is implemented in a structured and culturally sensitive manner, it reduces decisional conflict, facilitates complex end-of-life conversations, and results in care that is more consistent with the patient’s goals (Meier et al., 2016; Rietjens et al., 2017).

When these conversations included family members and addressed topics such as priorities, beliefs, legacy, or life closure tasks, patients experienced greater clarity, relief, and security regarding their decisions (García-Navarro et al., 2021; Kukla et al., 2022; Tobin et al., 2022; Wisesrith et al., 2021). The distinctive feature of ACP was the accessible, up-to-date documentation of preferences, which enabled better coordination between services, avoided unwanted interventions, and ensured continuity of care (Meier et al., 2016; Rietjens et al., 2017).

Although barriers such as lack of training, limited time, and the absence of standardized institutional systems persisted (Al-Ghabeesh et al., 2018; Tirgari et al., 2022), the studies agreed that integrating PCA into routine practice strengthened autonomy, dignity, and coherence of care in advanced illnesses. In this way, ACP not only incorporates the spiritual dimension but also operationalizes it, ensuring that the person’s values and desires honestly guide decisions at the end of life.

These findings directly addressed the review objective, demonstrating how spirituality influences well-being while also outlining practical avenues for its integration into person-centered care (Table 3).

**Table 3:** Alignment of review objectives with main findings on the influence of spirituality in advanced illness and end-of-life care

Study objective	Key findings	Interpretation / Implications for care
1. Examine how spirituality influences coping, relief of suffering, and emotional well-being in patients with advanced illness or at the end of life	Spirituality enhances coping through meaning, hope, and resilience Reduces emotional and existential distress, promoting acceptance of dying Improves quality of life and feelings of inner peace and connection	Spirituality functions as a determinant of health, not a comfort measure. Integrating meaning and peace fosters psychological adaptation and emotional stability in serious illness
2. Identify the mechanisms through which spiritual care contributes to holistic well-being	Effective mechanisms include presence, empathy, relational connection, and value-concordant decision-making Meaning/Peace dimension yields the most consistent improvements in outcomes	The Meaning/Peace pathway is the most robust mechanism, confirming that spirituality strengthens resilience and supports dignified end-of-life experiences
3. Explore barriers to integrating spirituality into clinical practice	Persistent confusion between spirituality and religiosity Limited professional training and scarcity of validated assessment tools Lack of institutional infrastructure to support implementation	Barriers indicate the need for standardized training programs, validated tools, and organizational commitment to integrate spirituality as a clinical competency
4. Identify opportunities and strategies for implementation in holistic, person-centered care	Advance Care Planning translates spiritual assessment into actionable decisions Interdisciplinary collaboration, chaplaincy inclusion, and cultural adaptation enhance sustainability	Embedding spirituality in ACP and team-based care promotes dignity, autonomy, and cultural congruence, advancing person-centered models of serious illness care

Table 3 aligns the review objectives with key findings, highlighting how spirituality influences well-being and summarizing emergent themes, such as barriers and opportunities, that enrich understanding of its integration in palliative care

ACP, Advance care planning

## Discussion

This integrative review addressed the central question: How, and through which mechanisms, does spiritual care influence the well-being of patients with advanced chronic illness or those nearing the end of life? Across diverse methodologies, the findings demonstrated that intentional integration of meaning, inner peace, relational connection, and value-based decision-making was associated with reduced emotional distress, greater resilience, and deeper acceptance of the dying process. Taken together, these results confirm that spirituality functions as a determinant of health, rather than as an optional source of comfort, thereby shifting contemporary healthcare toward a more holistic paradigm beyond the traditional biomedical model (Bernard et al., 2017; Jaman-Mewes et al., 2024; Kruizinga et al., 2019; Rego et al., 2020).

This review filled a significant gap by synthesizing heterogeneous evidence—quantitative, qualitative, and implementation-focused—to propose an operational model that translates spiritual assessment into concrete clinical actions (Khouzani et al., 2025). The model integrates three interconnected components: (a) assessment of spiritual needs, (b) structured conversations centered on meaning, and (c) documentation of values and preferences within Advance Care Planning (ACP). This synthesis bridges the longstanding divide in the literature between clinical outcomes research and practical models that guide real-world implementation.

Our findings align with those of Coelho et al. (2025), Dallı et al. (2025) and Long et al. (2024), who argue that spirituality should be recognized as a determinant of health and operationalized through professional competence, training, and institutional commitment. While Long et al. emphasize system-level and policy frameworks, our review provides empirical evidence of a direct link between spirituality and improved patient outcomes. It highlights the need to professionalize spiritual care through training, leadership engagement, and structured documentation.

Similarly, the work of Meeprasertsagool et al. (2025) in Thailand supports our findings by demonstrating that structured spiritual-care models—grounded in staff training, systematic assessment, and interdisciplinary collaboration—can be effectively implemented even in settings with limited specialized providers. Our review expands this perspective by showing how these components interact with value-based decision-making to enhance the alignment of care in advanced illness.

The study by Wehbeh et al. (2025) in Lebanon further reinforces the importance of cultural sensitivity, illustrating that spiritual care tools developed in Western contexts are not always appropriate in regions with distinct religious, cultural, or political dynamics. Consistent with our findings, these authors highlight the need to adapt spiritual assessment and interventions to local belief systems to preserve trust, meaning, and therapeutic connection.

Additionally, Gonçalves Júnior et al. (2025) demonstrated that spirituality and religiosity served as protective factors against stress and hopelessness in individuals with rheumatic diseases. Their results resonate with our conclusion that spirituality—particularly meaning, connection, and coherence—enhances resilience across different chronic illness populations.

### Limitations and Strengths

This integrative review provided a broad understanding of how spirituality influences health across the advanced illness trajectory. Although heterogeneity in methods, measurement tools, and predominantly Western samples limited comparability and introduced interpretive subjectivity, the synthesis provided a solid foundation for future research. It supported the development of a model of spiritual well-being.

### Clinical, Practical, and Educational Implications

This review highlights the importance of integrating spirituality as a fundamental component of care for individuals with advanced or terminal illnesses. Building spiritual-care competencies, such as empathetic communication, identifying needs, and referral, enhances well-being and shared decision-making, leading to more compassionate, person-centered, and dignified care across all care settings.

In addition, the findings of this review offer practical and educational implications that complement the clinical considerations described above. Practically, the proposed model highlights core competencies that can be taught and applied across disciplines: identifying spiritual needs, facilitating meaning- and values-based conversations, and documenting preferences within ACP. These skills can be integrated into brief trainings, clinical guidelines, and institutional protocols to strengthen shared decision-making and care alignment. Educationally, incorporating spiritual care content into undergraduate and postgraduate curricula, using simulation-based training to enhance communication skills, and promoting interprofessional education would help clinicians develop confidence, cultural sensitivity, and the ability to address spiritual and existential distress in a systematic and dignified manner.

### Implications for Future Research

Future research should develop brief, culturally sensitive instruments that distinguish spirituality from psychological constructs to avoid scale contamination (Koenig & Carey, 2024), test scalable interventions such as meaning-centered conversations or ACP in diverse populations, and use implementation-oriented designs to evaluate feasibility, adoption, and cost-effectiveness, thereby informing clinical practice and policy.

## Conclusions

This integrative review demonstrated that spiritual care, implemented through meaning, peace, connection, and values, was associated with reduced anxiety and depression, increased hope and resilience, and improved quality of life in people with advanced chronic illness and at the end of life. Therefore, spirituality emerges as an essential clinical component that can be implemented in three simple steps: a brief universal screening with clear referral pathways; structured, meaning-focused conversations (with family involvement); and integration with ACP through accessible and up-to-date documentation. These findings inform the design of future studies, incorporating culturally sensitive measures and trials of brief, meaning-focused interventions, while driving immediate changes in practice and supporting policies that recognize spiritual care as a patient’s right and a quality standard. This promotes values-consistent decisions and a more humane and dignified end-of-life experience.

## Data Availability

The data supporting this study’s findings are available from the corresponding author upon reasonable request.

## Code Availability

It is not possible to provide code because the work is based on the review and analysis of data from existing studies, not on original software or an algorithm.
